# Antipsychotics and Mortality in Adult and Geriatric Patients with Schizophrenia

**DOI:** 10.3390/ph17010061

**Published:** 2023-12-29

**Authors:** Ling-Ling Yeh, Wei-Chen Lee, Kuei-Hong Kuo, Yi-Ju Pan

**Affiliations:** 1 Graduate School of Humanities and Social Sciences, Dharma Drum Institute of Liberal Arts, New Taipei City 208, Taiwan; yehll@dila.edu.tw; 2 Department of Psychiatry, Far Eastern Memorial Hospital, New Taipei City 220, Taiwan; 101311144@gms.tcu.edu.tw; 3 Division of Medical Imaging, Far Eastern Memorial Hospital, New Taipei City 220, Taiwan; 4 School of Medicine, National Yang Ming Chiao Tung University, Taipei 112, Taiwan; 5 Department of Chemical Engineering and Materials Science, Yuan Ze University, Taoyuan City 320, Taiwan

**Keywords:** antipsychotic, mortality, schizophrenia, older adult, daily defined dosage

## Abstract

Patients with schizophrenia have a high mortality risk, and the role of antipsychotic medications remains inconclusive. In an aging society, older patients with schizophrenia warrant increased attention. This study investigated the association of antipsychotic medication dosages with mortality in patients with schizophrenia by using data from Taiwan’s National Health Insurance Research Database from 2010 to 2014. This study included 102,964 patients with schizophrenia and a subgroup of 6433 older patients in addition to an age- and sex-matched control group. The findings revealed that among patients with schizophrenia, the no antipsychotic exposure group had the highest mortality risk (3.61- and 3.37-fold higher risk for overall and cardiovascular mortality, respectively) in the age- and sex-adjusted model, followed by the high, low, and moderate exposure groups. A similar pattern was observed in the older patients with schizophrenia. High exposure to antipsychotics was associated with the highest risks of overall and cardiovascular mortality (3.01- and 2.95-fold higher risk, respectively). In conclusion, the use of antipsychotics can be beneficial for patients with schizophrenia with recommended exposure levels being low to moderate. In older patients, high antipsychotic exposure was associated with the highest mortality risk, indicating that clinicians should be cautious when administering antipsychotic medications to such patients.

## 1. Introduction

Compared with the general population, individuals with schizophrenia have a substantially higher risk of mortality [1,2,3]. In Taiwan, individuals with schizophrenia have a life expectancy at birth that is approximately 15 years shorter than that of the general population [4]. This finding is consistent with the literature [5,6]. Individuals with severe mental illness have a high risk of death from unnatural causes; however, approximately three-fourths of all deaths among such individuals are classified as occurring due to natural causes [1], and research indicates that a sedentary lifestyle, obesity, limited access to health services, and the adverse effects of medications [7,8,9,10] may contribute to this population’s high mortality rates.

Increased mortality in patients with schizophrenia may be attributable to the adverse effects of antipsychotic medications, which include weight gain, metabolic syndrome, diabetes, and ischemic heart disease [11,12]. A pharmaco-epidemiological study reported a dose-related risk of sudden cardiac death among patients using antipsychotic medications [13]. However, several studies have been conducted using large, prospectively collected datasets of actual filled prescriptions to investigate the risk of death associated with any, current, or cumulative antipsychotic exposure in patients with schizophrenia, and their results have indicated that the use of an antipsychotic is associated with a lower risk of mortality than nonuse of an antipsychotic is [5,14,15,16]. Furthermore, studies have revealed a U-shaped relationship between antipsychotic exposure and overall mortality, indicating that low and moderate levels of antipsychotic exposure are associated with a substantially lower risk of mortality than no or high exposure are [17,18]. However, evidence is lacking regarding the association between mortality and antipsychotic exposure relative to the mortality of a control group without psychiatric diagnoses with consideration of socioeconomic factors and comorbid physical conditions.

With the advancement of health-care services, the average life expectancy has gradually increased. In many developed countries, individuals aged ≥55 years will soon account for a quarter or more of the population with schizophrenia [19]. Whiteford et al. reported that schizophrenia currently ranks third among psychiatric disorders in terms of causes of disability-adjusted life years for people aged 60 years or older [20]. In addition, older patients with schizophrenia have a high prevalence of comorbid medical conditions [21], which may influence their use of psychotropic medications and their risk of mortality. However, research on older individuals with schizophrenia is limited, accounting for only approximately 1% of the literature on schizophrenia [22], and the association between exposure to psychotropic medications and mortality in the geriatric population remains under-researched.

This study investigated the association between the degree of cumulative antipsychotic exposure, as indicated by the number of filled prescriptions, and mortality in a national cohort of patients with schizophrenia, with the risk of mortality in this population compared with that in the general population (control group). In addition, this study analyzed the association between mortality and cumulative antipsychotic doses in a subgroup of older individuals with schizophrenia.

## 2. Results

Table 1 lists the demographic and clinical characteristics of the participants. In total, 102,964 patients with schizophrenia were enrolled. The mean age of the schizophrenia cohort was 44.8 (SD = 13.2) years, and 47.4% of the patients were women. The subgroup of older patients with schizophrenia comprised 6433 individuals with a mean age of 73.6 (SD = 6.7) years, and 59.7% of these patients were women. Compared with the corresponding control sample, the patients with schizophrenia had higher rates of chronic obstructive pulmonary disease (COPD), cardiovascular diseases (CVDs), diabetes mellitus (DM), and renal disease (RD). However, in the older patient subsample, compared with the corresponding control sample, the older patients with schizophrenia had higher rates of only COPD and CVD. In terms of mortality, 7730 patients with schizophrenia (7.5%) and 2593 controls (2.5%) died during the 5-year follow-up period. The mortality rate in the older patients with schizophrenia (31.9%) was approximately twice that observed in the corresponding control sample during the 5-year follow-up period.

For the patients with schizophrenia, 8733 (8.5%) had no antipsychotic exposure during the follow-up period, whereas 19,017 (18.5%) had high antipsychotic exposure (Table 2). Among the 6433 older patients with schizophrenia, 944 (14.7%) had no antipsychotic exposure during the follow-up period, whereas 3520 (54.7%) and 333 (5.2%) had low and high antipsychotic exposure, respectively.

**Figure 1:**
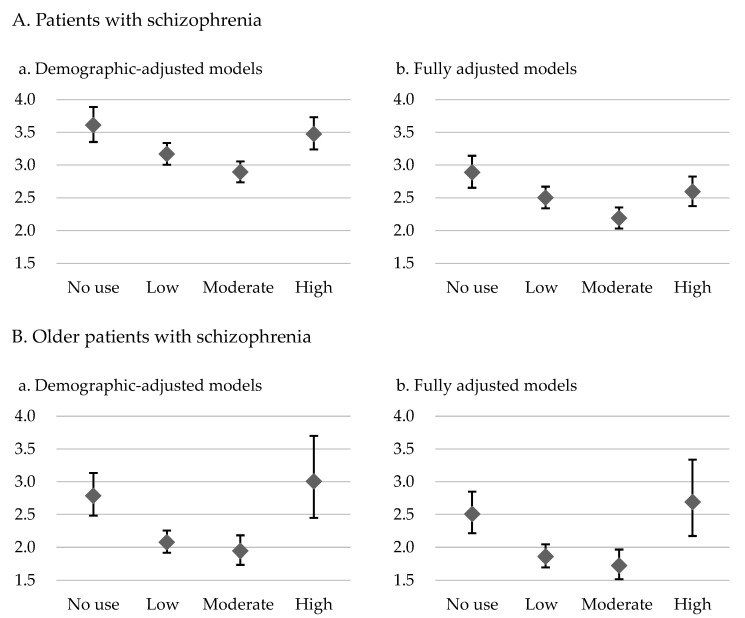
Overall mortality hazard ratios and 95% confidence intervals for level of exposure to antipsychotics in the demographic-adjusted model (**a**) and fully adjusted model (**b**) for patients with schizophrenia and older patients with schizophrenia relative to controls without psychiatric diagnoses.

**Figure 2:**
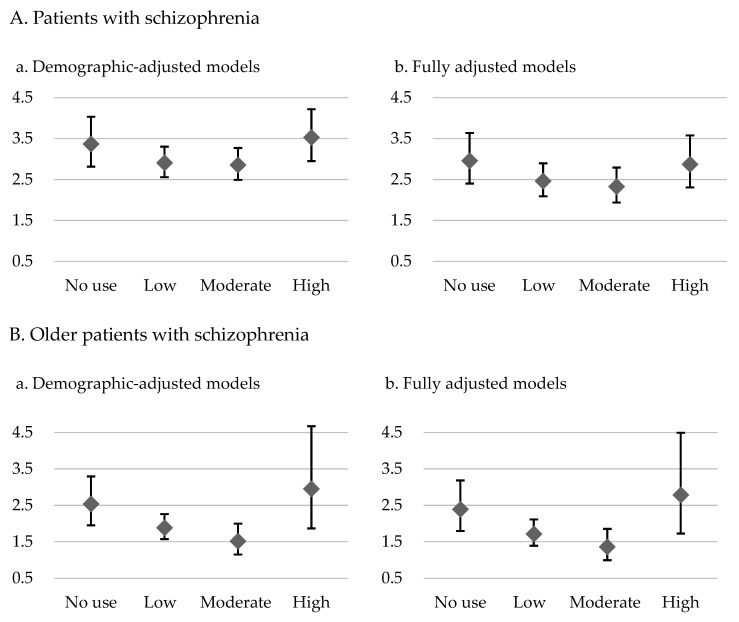
CVD-related mortality hazard ratios and 95% confidence intervals for level of exposure to antipsychotics in the demographic-adjusted model (**a**) and fully adjusted model (**b**) for patients with schizophrenia and older patients with schizophrenia relative to controls without psychiatric diagnoses.

The results for the demographic-adjusted model (model 1) and the fully adjusted model (model 2) are presented in Table 2 and Table 3, respectively. The patients with schizophrenia who had no exposure to antipsychotics had 3.61-fold (model 1) and 2.89-fold (model 2) higher risks of overall mortality and 3.37-fold (model 1) and 2.96-fold (model 2) higher risks of CVD-related mortality than did the control group. In the demographic-adjusted and fully adjusted models, U-shaped curves were noted for the associations of antipsychotic exposure with overall and CVD-related mortality risk (Table 2 and Table 3; Figure 1 and Figure 2). Among the patients with schizophrenia, the no exposure group had the highest overall mortality risk (model 1: HR = 3.61, 95% CI: 3.35–3.89; model 2: HR = 2.89, 95% CI: 2.65–3.14), followed by the high, low, and moderate exposure groups, despite some of the confidence intervals overlapping. Furthermore, the no exposure group had the highest CVD-related mortality risk (model 1: HR = 3.37, CI: 2.82–4.04; model 2: HR = 2.96, 95% CI: 2.40–3.64), followed by the high, low, and moderate exposure groups.

For the older patients with schizophrenia, U-shaped associations of level of exposure to antipsychotics with overall mortality and CVD-related mortality were noted (Table 2 and Table 3; Figure 1 and Figure 2). Furthermore, the high exposure group had the highest overall mortality risk (model 1: HR = 3.01, 95% CI: 2.45–3.70; model 2: HR = 2.69, 95% CI: 2.17–3.34), followed by the no, low, and moderate exposure groups, despite some of the confidence intervals overlapping. A similar pattern was observed for CVD-related mortality in these patients. The high exposure group had the highest CVD-related mortality risk (model 1: HR = 2.95, CI: 1.87–4.67; model 2: HR = 2.78, 95% CI: 1.72–4.50), followed by the no, low, and moderate exposure groups.

## 3. Discussion

This study investigated the association between cumulative exposure to antipsychotics and excess mortality in patients with schizophrenia through a comparison with an age- and sex-matched control group. In addition, we analyzed such an association in a subgroup of older patients with schizophrenia. The results revealed U-shaped associations of exposure to antipsychotics with overall and CVD-related mortality in the patients with schizophrenia; the highest risk of mortality was observed in the patients with no antipsychotic exposure, whereas the lowest risk of mortality was noted in those with low to moderate antipsychotic exposure. In the older individuals with schizophrenia, U-shaped associations of antipsychotic exposure with overall and CVD-related mortality were also noted. However, in this subgroup, high antipsychotic exposure was associated with the highest risks of overall and CVD-related mortality. These findings highlight the importance of using an adequate dosage of antipsychotic medications for patients with schizophrenia. The finding of an association between a high antipsychotic dosage and increased risk of mortality in older patients with schizophrenia highlights the need for clinicians to remain vigilant when adjusting antipsychotic dosages for these patients.

The no exposure group had an age- and sex-adjusted (model 1) HR of 3.61 and a fully adjusted (model 2) HR of 2.89 for overall mortality and an age- and sex-adjusted (model 1) HR of 3.37 and a fully adjusted (model 2) HR of 2.96 for CVD-related mortality compared with the control group. After socioeconomic factors and physical comorbidities were adjusted for, the mortality rate in model 2 was lower. Seeman reported that poor socioeconomic status is a barrier to reducing the mortality gap between individuals with schizophrenia and the general population [23]. In addition, studies have indicated that patients with schizophrenia have a higher prevalence of comorbid physical conditions, such as diabetes, COPD, and CVD, than do those without schizophrenia [16,24,25,26]. The higher mortality in patients with schizophrenia might be attributable to the presence of comorbidities, particularly CVD [26]; undetected or inadequately treated comorbid physical illnesses can lead to an increased mortality risk [8,16]. Our results revealed that the patients with schizophrenia with no antipsychotic exposure had an approximately three-fold higher risk of overall and CVD-related mortality than the control group did, even after socioeconomic variables and comorbid physical illnesses were controlled for (model 2). A systematic review including 135 studies published spanning from 1957 to 2021 reported a 2.9-fold higher risk of all-cause mortality in patients with schizophrenia than in the general population and a 1.6-fold higher risk in patients with schizophrenia than in physical disease–matched controls from the general population [27]. It seems likely that schizophrenia per se may be independently associated with an elevated overall and CVD-related mortality, as presented in the current findings for the patients with no antipsychotic exposure. Several symptoms of schizophrenia could lead to a higher mortality risk in individuals with the disorder. For example, lack of insight is a symptom of schizophrenia [28], as indicated by the finding of the WHO’s 10-country study on schizophrenia that 98% of patients with schizophrenia exhibit a lack of insight, making it the most prevalent symptom [29]. Lack of insight is the primary cause of treatment nonadherence [30]. Moreover, various interconnected factors in schizophrenia might synergistically contribute to physical morbidity and subsequent mortality in patients. Peritogiannis et al. collected and classified these factors, including patient-related factors, symptomatology, treatment-related factors, health service–related factors, other disease–related factors, and socioeconomic factors [31]. Patient-related factors such as unhealthy lifestyle, including smoking, substance use, alcohol use, sedentary lifestyle, and poor nutritional habits, contribute to higher mortality due to natural causes in patients with schizophrenia [31]. In particulary, with smoking, a study reported that approximately 70–80% of patients with schizophrenia were smokers and smoked a higher number of cigarettes than did those smokers without schizophrenia [32,33]. The life expectancy for smokers is at least 10 years shorter than that for nonsmokers [34,35], and smoking is causally associated with mortality in various CVDs [36,37]. The high mortality risk in patients with schizophrenia may also be linked to societal factors and the characteristics of the health-care system [31]. Evidence indicates that patients with schizophrenia and physical comorbidities may not receive appropriate medical care. A Danish study reported that patients with schizophrenia and heart failure received lower-quality care that deviated from guidelines compared with that received by patients without schizophrenia with heart failure. Notably, inadequate psychosocial functioning in schizophrenia patients predicted suboptimal heart failure care, leading to a substantially higher 1-year mortality risk [38]. Not only do they accept improper medical care, but patients with schizophrenia are also faced with discrimination or have some kind of stigma that may negatively affect their health. A previous cross-sectional survey conducted across 27 countries revealed that more than 17% of patients with schizophrenia encountered discrimination when receiving treatment for physical health problems. Perceived discrimination may deter patients with schizophrenia from seeking medical services, thereby contributing to less favorable outcomes in addressing their physical health issues [39]. Therefore, it is crucial for clinicians to adequately address the aforementioned habits and behaviors associated with patients with schizophrenia, as well as the relevant social-environmental factors.

In this study, U-shaped associations of antipsychotic exposure with overall and CVD-related mortality in the patients with schizophrenia were observed, with these findings indicating that the risk of mortality may be highest among patients with no antipsychotic exposure (Figure 1 and Figure 2). However, the confidence intervals were overlapping across certain groups, which necessitates a more cautious interpretation. For instance, as presented in Table 2, we found that schizophrenia patients of the low dose and moderate dose groups had lower overall mortality compared to those in the no use and high dose groups, with non-overlapping confidence intervals, whereas the confidence intervals were overlapping between the no use and high dose groups. In a Finnish study involving more than 60,000 patients treated for schizophrenia in inpatient settings and with a follow-up of 20 years, compared with nonuse, any antipsychotic use was associated with lower all-cause mortality (HR = 0.48) [40]. Additionally, Crump et al. revealed that nonuse of antipsychotics was associated with elevated mortality [16]. However, both of these studies compared use and nonuse of antipsychotic medications without considering the effects of different antipsychotic doses. Torniainen et al. investigated the associations between antipsychotic exposure and mortality while also examining the effects of varying antipsychotic doses [18]. Our results are comparable to their study, revealing a U-shaped association between overall mortality and different levels of cumulative antipsychotic exposure [18]. However, the aforementioned study did not concurrently account for the influence of socioeconomic factors on mortality or consider the potentially high prevalence of comorbid physical illnesses in patients with schizophrenia. Besides overall mortality, we found a similar U-shaped pattern for CVD-related mortality in the present study. A 24-year national register study conducted in Sweden revealed that CVD was the leading cause of mortality among patients with schizophrenia. In addition, it revealed that patients with schizophrenia generally experienced CVD-related mortality 10 years earlier than the general population did [41]. In contrast to the general population, individuals with schizophrenia exhibited elevated and earlier CVD-related mortality. This disparity can be attributed to multiple factors, encompassing patients’ comorbidities, harmful health-related behaviors, social factors such as stigma, insufficient preventive interventions, limited health literacy, suboptimal adherence to essential management, barriers to health-care access, and a propensity for accepting suboptimal care [42,43]. Antipsychotic medications, as the primary pharmacotherapy for treating schizophrenia, have been extensively studied for their efficacy in addressing psychiatric symptoms and potential cardiovascular complications. Adequate exposure to antipsychotic treatment is crucial for patients with schizophrenia. Nonadherence to antipsychotic medication is linked to several psychiatric symptoms, poorer mental functioning, poorer life satisfaction, and increased substance use [44]. These problems may worsen the health condition of patients with schizophrenia. However, the literature has revealed that antipsychotics, regardless of whether they are first- or second-generation antipsychotics, are associated with metabolic and cardiovascular side effects [45,46,47]. Another study reported that antipsychotics were associated with a dose-related increase in the risk of sudden cardiac death [13]. These findings indicate that the use of antipsychotic medications warrants attention because they may increase the risks of CVD and associated mortality. However, based on previous research, especially clinical database studies, the risk of CVD-related mortality may not exhibit a positive correlation with the use of antipsychotic medications. For example, Torniainen et al. reported a U-shaped association between cumulative antipsychotic exposure and CVD-related mortality [18], a finding that is comparable to our own. Another crucial issue is when, or if, stable patients with schizophrenia can reduce their antipsychotic medication dosage. A review published in 2022 collected evidence on the reduction of antipsychotic doses for stable individuals with schizophrenia. The review revealed no difference between the groups undergoing dose reduction and those continuing the same dose with regard to quality of life, functioning, and the incidence of participants experiencing at least one adverse effect. However, the dose reduction group exhibited an increased susceptibility to relapse, dropping out, and rehospitalizations [48]. These findings highlight the importance of achieving a balance in prescribing antipsychotic medications, that is, in prescribing adequate doses that effectively treat symptoms, enhance patients’ quality of life, and enable management of side effects.

In this study, we observed that compared with the corresponding control group, the older patients with schizophrenia who had no antipsychotic exposure had 2.79-fold (model 1) and 2.51-fold higher risks (model 2) of overall mortality and 2.54-fold (model 1) and 2.39-fold higher risks (model 2) of CVD-related mortality. The literature indicates that patients with schizophrenia have a higher risk of mortality that continues into old age, although the mortality gap is smaller in older individuals than in younger ones [49,50]. A survivor effect might explain these findings; the patients who remained alive throughout the study period might have been healthier than those who died were. Although the mortality gap between the older patients with schizophrenia and the control group was smaller than that observed between the younger patients with schizophrenia and the control group, the health status of older patients with schizophrenia warrants greater attention. Research has indicated that compared with healthy individuals, older patients with schizophrenia receive less adequate treatment for physical comorbidities [51,52]. Inadequate treatment may result in compromised health and diminished quality of life in older patients with schizophrenia, which can lead to an increased medical and societal burden. In the context of an aging population, the vulnerability of older patients with schizophrenia must be considered, and clinicians must ensure that such patients receive adequate treatment for physical comorbidities.

In the current study, the older patients with schizophrenia had the highest risk of overall and CVD-related mortality in the high antipsychotic exposure group (Figure 1 and Figure 2), although the confidence intervals were overlapping across certain groups. Table 2 revealed that among the older patients with schizophrenia, those in the low and moderate exposure groups had lower overall mortality than did those in the no and high exposure groups, with nonoverlapping confidence intervals. However, for CVD mortality, the confidence intervals overlapped across the groups with different exposure levels, despite the illustrated U-shaped curves. The side effects of antipsychotics, which can affect all patient populations, may be particularly pronounced in older patients, because age-related changes can amplify these effects [53]. For example, age-related changes in hepatic and renal functions markedly affect the absorption, distribution, metabolism, and excretion of drugs. Liver mass, hepatic blood flow, serum albumin levels, and renal blood flow and function tend to decrease with age [54]. In addition, age-related alterations in body composition can affect the pharmacokinetics of antipsychotics in older patients. These changes may involve a decrease in lean muscle mass and total body water and an increase in total body fat [53]. Moreover, because of age-related increases in monoamine oxidase activity, in older patients, the central nervous system exhibits heightened sensitivity to antipsychotic drugs. In addition, a reduction in cerebral blood flow and a selective decline in some nerve pathways were reported in older patients. Furthermore, the age-related loss of cholinergic neurons and the exacerbation of cholinergic deficits by these drugs increased the sensitivity of older patients to medications exerting anticholinergic effects [54]. Older patients with schizophrenia may have comorbid chronic diseases, such as CVD and diabetes, and antipsychotic use may increase the difficulty of managing these diseases [53]. A study revealed that both first- and second-generation antipsychotics were associated with an increase in the risk of mortality among older patients [55], and dosage may be a key determinant of antipsychotic safety, which affects mortality risk [56]. In summary, low or moderate doses of antipsychotics may be sufficient for treating older patients with schizophrenia, and as patients advance in age and their physical health condition changes, clinicians should adjust the dosage of antipsychotic medications. For older patients with schizophrenia, high exposure to antipsychotic medications is not generally recommended. If high doses of antipsychotic medication are required, clinicians must closely monitor for associated side effects and potential risk factors.

The strengths of this study include nationwide coverage, encompassing patients diagnosed with schizophrenia in all clinical settings, which has benefits in increasing the generalizability of the results, and comparisons with a control sample without psychiatric diagnoses. However, this study has several limitations. First, due to its non-randomized study design, we needed to be cautious when interpreting the results due to potential selection bias. For instance, certain characteristics of the no exposure group, such as lack of connection to health-care systems, may confound the presented associations between dose exposure and mortality. Second, the observational nature of the study may limit its ability to establish causal relationships. Third, the lack of accurate information regarding disease severity and patient lifestyles in the NHIRD limited our assessment of these factors, which can affect mortality. Fourth, because the data were mainly obtained from the NHIRD, the current results cannot be directly generalized to populations with different characteristics and under different health-care systems without proper adjustment. Finally, we did not directly measure the actual amount of medications received by the patients or their blood levels of antipsychotic medications.

## 4. Materials and Methods

### 4.1. Setting

This study was approved by the Research Ethics Review Committee of Far Eastern Memorial Hospital in Taiwan (109150-E). Taiwan has a population of approximately 23 million, and its National Health Insurance system is a compulsory, single-payer health-care system. In this system, the disbursement of funds is centralized, and all Taiwanese citizens and legally employed foreign workers in Taiwan have equal access to health-care services. The National Health Insurance Research Database (NHIRD) contains comprehensive records of the health service utilization of nearly the entire Taiwanese population; these records include information regarding demographics, procedures, and medication usage and corresponding medical service expenditures. From its inception until 2016, disease data in the NHIRD were coded using the International Classification of Diseases, Ninth Revision, Clinical Modification (ICD-9-CM) [57].

### 4.2. Study Population

**Figure 3:**
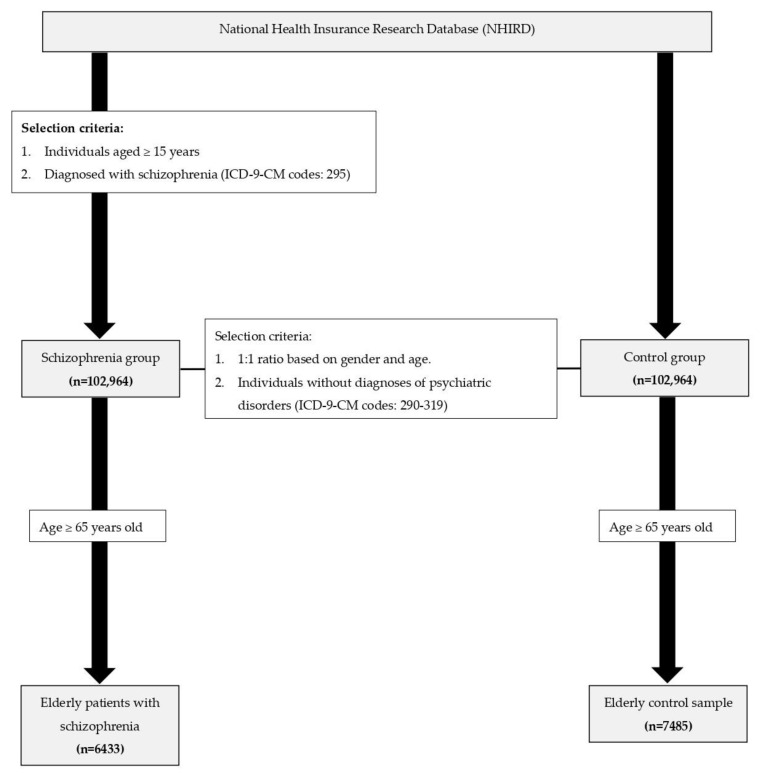
Flowchart for patient selection and recruitment.

In the present study, individuals with schizophrenia were identified on Taiwan’s NHIRD, which is managed by the Health and Welfare Data Science Center of the Ministry of Health and Welfare in Taiwan. We identified and included individuals aged ≥15 years who were given a diagnosis of schizophrenia (ICD-9-CM code 295) in 2010. These patients were followed up on for 5 consecutive years (2010–2014), which served as the observation window. The study cohort comprised patients with both incident and existing cases with schizophrenia. In addition, this study identified a subgroup of older patients with schizophrenia who were aged ≥65 years in the index year. The mortality in the patients with schizophrenia was compared with that in a control sample. The control sample was randomly selected from the Registry for Beneficiaries of the NHIRD and was age- and sex-matched with the patients with schizophrenia. We selected individuals without diagnoses of psychiatric disorders (ICD-9-CM codes 290–319) from the registry and then matched them by sex. We subsequently randomly selected individuals from the control sample for age matching based on 10-year age intervals (15–20, 21–30, 31–40, 41–50, 51–60…, 91–100, and >100 years). For the subgroup analysis of older patients, a control sample of individuals aged ≥65 years was selected from the overall control sample. The flowchart for patient selection and recruitment is illustrated in Figure 3. As for mortality, we identified the causes of death through linkage to Taiwan’s national mortality registry. In addition to overall mortality, we analyzed the number of deaths due to cardiovascular diseases (CVDs, ICD-9-CM codes I00–I99).

### 4.3. Covariates

Data on age, sex, socioeconomic variables, possession of a catastrophic illness certification, and diagnoses of physical illnesses were extracted for both the patients with schizophrenia and the control sample. The following socioeconomic variables were analyzed: household income, urbanization level of residence, and insurance premium level (determined on the basis of the monthly income of the insured). The investigated covariates related to physical illnesses included diagnosed chronic obstructive pulmonary disease (COPD), CVD, cancer, diabetes mellitus (DM), and renal disease (RD). In addition, for the patients with schizophrenia, we extracted treatment-related data, including psychiatric and nonpsychiatric health-care costs, psychiatric ward admission records, and antipsychotic medication dosage information. The antipsychotic medications considered in our study were aripiprazole, amisulpride, clozapine, olanzapine, quetiapine, risperidone, ziprasidone, zotepine, paliperidone, chlorpromazine, haloperidol, fluphenazine, and thioridazine. The mean defined daily dose (DDD) is the recommended average daily maintenance dose of a drug used for its main indication in adults; the present study referenced the DDD guidelines of the World Health Organization (WHO) [58]. We calculated the mean DDD of antipsychotics by dividing the cumulative dose by the number of follow-up days. Subsequently, we categorized the patients into four groups on the basis of their exposure to antipsychotic medications: no exposure, low exposure (<0.5 DDD), moderate exposure (0.5–1.5 DDD), and high exposure (>1.5 DDD).

### 4.4. Statistical Analyses

We first compared the demographic and socioeconomic characteristics between the patients with schizophrenia and the control group and between the older patients with schizophrenia and the corresponding control group. Categorical variables were analyzed using the chi-squared test, whereas continuous variables were analyzed using F tests. Cox regression analysis was performed to compare the overall mortality and CVD-related mortality among the different antipsychotic exposure groups relative to those of the control group. Two regression models were used. The first model included only age and sex as covariates (demographic-adjusted model), and the second included sex; age; possession of a catastrophic illness certification; socioeconomic variables, including insurance premium level and household income; and presence of comorbid physical illnesses, such as COPD, CVD, cancer, DM, and RD, included as covariates (fully adjusted model). Hazard ratios (HRs) for overall mortality were calculated for the exposure groups (i.e., the no exposure, low exposure, moderate exposure, and high exposure groups). HRs for CVD-related mortality were also calculated for the exposure groups. Statistical significance was set at *p* < 0.05. All statistical analyses were performed using SPSS, version 21.0 (IBM, Armonk, NY, USA).

## 5. Conclusions

In this study, we discovered that although the higher mortality rates in the patients with schizophrenia were partly attributable to their comorbidities, schizophrenia was independently associated with an increase in overall and CVD-related mortality. The use of antipsychotics was beneficial to the patients with schizophrenia, and the most recommended dosage was low to moderate. In the older patients with schizophrenia, high antipsychotic exposure was associated with the highest mortality risk. The findings of this study indicate that improving drug adherence among patients with schizophrenia is crucial. In addition, given that research on older patients with schizophrenia is limited, this study provides valuable insights, indicating that an appropriate dose of antipsychotic medications must be prescribed in older patients with schizophrenia to prevent adverse outcomes related to high exposure to antipsychotic medications. Future studies should investigate the mechanisms underlying the associations between antipsychotic dosage and mortality in different age groups of patients with schizophrenia.

## Figures and Tables

**Table 1:** Demographic characteristics of patients with schizophrenia (n = 102,964), older patients with schizophrenia (n = 6433), and control samples.

	Patients with Schizophrenia (n = 102,964)	Control Sample (n = 102,964)	Significance	Elderly Patients with Schizophrenia (n = 6433)	Control Sample (n = 7485)	Significance
Age (years old) [mean (SD)]	44.8 (13.2)	44.8 (13.6)	*F* = 720.946 **	73.6 (6.7)	72.9 (6.5)	*F* = 40.762 **
Gender [n (%)]			*χ*^2^ = 0.0 **			*χ*^2^ = 0.079
Female	48,813 (47.4)	48,813 (47.4)		3843 (59.7)	4489 (60.0)	
Male	54,151 (52.6)	54,151 (52.6)		2590 (40.3)	2996 (40.0)	
Lower-income household [n (%)]	13,129 (12.8)	778 (0.8)	*χ*^2^ = 107,017.511 **	948 (14.7)	58 (0.8)	*χ*^2^ = 1005.687 **
With catastrophic illness certificate ^I^ [n (%)]	74,540 (72.4)	2673 (2.6)	*χ*^2^ = 107,017.511 **	4049 (62.9)	602 (8.0)	*χ*^2^ = 4686.137 **
Chronic diseases						
COPD [n (%)]	8796 (8.5)	5055 (4.9)	*χ*^2^ = 1083.264 **	1408 (21.9)	1085 (14.5)	*χ*^2^ = 128.549 **
CVD [n (%)]	10,575 (10.3)	9199 (8.9)	*χ*^2^ = 105.922 **	2151 (33.4)	2726 (36.4)	*χ*^2^ = 13.520 **
Cancer [n (%)]	1642 (1.6)	2165 (2.1)	*χ*^2^ = 73.202 **	309 (4.8)	503 (6.7)	*χ*^2^ = 23.136 **
DM [n (%)]	11,261 (10.9)	7001 (6.8)	*χ*^2^ = 1090.437 **	1477 (23.0)	1812 (24.2)	*χ*^2^ = 39.0484
RD [n (%)]	2414 (2.3)	2014 (2.0)	*χ*^2^ = 36.928 **	510 (7.9)	560 (7.5)	*χ*^2^ = 39.0484
Death [n (%)]			*χ*^2^ = 2691.217 **			*χ*^2^ = 517.351 **
All causes	7730 (7.5)	2593 (2.5)		2053 (31.9)	1168 (15.6)	
Natural causes	6176 (6.0)	843 (0.8)	*χ*^2^ = 695.821 **	1239 (19.3)	475 (6.3)	*χ*^2^ = 161.784 **
Cancer	1083 (1.1)	909 (0.88)		258 (4.0)	320 (4.3)	
CVD	1248 (1.2)	446 (0.43)		384 (6.0)	247 (3.3)	
DM	449 (0.4)	152 (0.15)		111 (1.7)	86 (1.1)	
Unnatural causes	1258 (1.2)	149 (0.14)		33 (0.5)	33 (0.4)	
Suicide	798 (0.8)	68 (0.07)		15 (0.2)	4 (0.1)	
Unknown	296 (0.3)	26 (0.03)		13 (0.2)	3 (0.0)	
Follow-up days [mean (SD)]	1735.52 (231.67)	1741.79 (174.7)	*F* = 261.113 **	1491.12 (529.14)	1655.51 (379.63)	*F* = 104.75 **

Continuous variables were compared using analysis of variance, and categorical variables were compared using a chi-squared test. ^I^ Individuals given a diagnosis by a physician of a condition classified as a catastrophic illness by the Ministry of Health and Welfare can apply for a catastrophic illness certificate, which exempts them from being required to make a copayment when receiving care for the specified illness. Abbreviations: SD, standard deviation; COPD, chronic obstructive pulmonary disease; CVD, cardiovascular disease; DM, diabetes mellitus; RD, renal disease. ** *p* < 0.001.

**Table 2:** Demographic (age and sex)-adjusted hazard ratios for different levels of exposure to antipsychotics in all patients and older patients with schizophrenia compared with controls (model 1).

	No Exposure		Low Exposure		Moderate Exposure		High Exposure	
	Adjusted Hazard Ratio	95% CI	Adjusted Hazard Ratio	95% CI	Adjusted Hazard Ratio	95% CI	Adjusted Hazard Ratio	95% CI
Patients with schizophrenia (n = 102,964) n (%)	8733 (8.5%)		33,403 (32.4%)		41,811 (40.6%)		19,017 (18.5%)	
Overall mortality	3.610	3.353–3.886	3.167	3.005–3.337	2.892	2.737–3.056	3.475	3.238–3.730
Cardiovascular mortality	3.370	2.815–4.035	2.907	2.559–3.304	2.856	2.492–3.272	3.529	2.952–4.218
Elderly patients with schizophrenia (n = 6433) n (%)	944 (14.7%)		3520 (54.7%)		1636 (25.4%)		333 (5.2%)	
Overall mortality	2.789	2.483–3.133	2.080	1.917–2.256	1.946	1.735–2.183	3.010	2.448–3.701
Cardiovascular mortality	2.536	1.952–3.295	1.885	1.572–2.259	1.513	1.148–1.996	2.953	1.865–4.674

Survival analysis was conducted using Cox regression with adjustment for sex and age (reference group = control group). Hazard ratios for overall and cardiovascular mortality were calculated for the no exposure (reference), low exposure (<0.5 DDD), moderate exposure (0.5–1.5 DDD), and high exposure (>1.5 DDD) groups. Abbreviations: CI, confidence interval; DDD, defined daily dose.

**Table 3:** Fully adjusted hazard ratios for antipsychotic exposure in patients with schizophrenia and older patients with schizophrenia relative to controls (model 2).

	No Exposure		Low Exposure		Moderate Exposure		High Exposure	
	Adjusted Hazard Ratio	95% CI	Adjusted Hazard Ratio	95% CI	Adjusted Hazard Ratio	95% CI	Adjusted Hazard Ratio	95% CI
Patients with schizophrenia (n = 102,964) n (%)	8733 (8.5%)		33,403 (32.4%)		41,811 (40.6%)		19,017 (18.5%)	
Overall mortality	2.888	2.654–3.142	2.500	2.340–2.671	2.187	2.032–2.354	2.591	2.375–2.826
Cardiovascular mortality	2.956	2.402–3.639	2.462	2.092–2.898	2.327	1.938–2.795	2.874	2.309–3.577
Elderly patients with schizophrenia (n = 6433) n (%)	944 (14.7%)		3520 (54.7%)		1636 (25.4%)		333 (5.2%)	
Overall mortality	2.511	2.214–2.849	1.862	1.696–2.044	1.727	1.517–1.965	2.692	2.172–3.337
Cardiovascular mortality	2.392	1.796–3.185	1.713	1.388–2.114	1.359	0.996–1.854	2.784	1.724–4.495

Survival analysis was conducted using Cox regression with adjustment for sex, age, possession of catastrophic illness certification, socioeconomic status (insurance premium level, household income, and urbanization level of residence), and comorbid physical illnesses (chronic obstructive pulmonary disease, cardiovascular disease, cancer, diabetes mellitus, and renal disease) (reference group = control group). Hazard ratios for overall and cardiovascular mortality were calculated for the no exposure, low exposure (<0.5 DDD), moderate exposure (0.5–1.5 DDD), and high exposure (>1.5 DDD) groups. Abbreviations: CI, confidence interval; DDD, defined daily dose.

## Data Availability

Available data are contained within the article.
